# Screening and functional validation of key genes in *Helicobacter Pylori*-induced macrophage M1 polarization: role in migraine-associated functional dyspepsia

**DOI:** 10.3389/fimmu.2026.1684555

**Published:** 2026-04-28

**Authors:** Nengjin Sun, Kaile Wang, Jianli Gou, Panpan Li, Xiaoyan Fu, Wenjing Shi, Jing Li, Miao Xiang, Shenglin Sun, Zihan Sun, Shujuan Liang, Yuying Zhang, Hongyan Wang

**Affiliations:** 1 Key Laboratory of Immune Microenvironment and Inflammatory Disease Research in Universities of Shandong Province, School of Basic Medical Sciences, Shandong Second Medical University, Weifang, China; 2 Department of Pathogenic Biology, School of Basic Medical Sciences, Shandong Second Medical University, Weifang, China; 3 Department of Gastroenterology, Weifang People’s Hospital, Shandong Second Medical University, Weifang, China; 4 School Hospital, Shandong Second Medical University, Weifang, China

**Keywords:** calcitonin gene-related peptide, functional dyspepsia, gut-brain axis, *Helicobacter pylori*, machine learning, macrophage, migraine

## Abstract

**Background:**

Migraine, a prevalent and debilitating neurovascular disorder, frequently co-occurs with functional dyspepsia. Emerging evidence suggests that *Helicobacter pylori (H. pylori)* infection may contribute to both conditions via neuroimmune pathways, though the molecular basis for this association has yet to be fully elucidated.

**Methods:**

*H. pylori*-related datasets from GEO were analyzed to identify differentially expressed genes (DEGs), followed by functional enrichment analyses including GO, KEGG, GSEA, and GSVA. Immune infiltration patterns were assessed, and CGRP-related hub genes were identified using machine learning approaches. The relationship between these hub genes and immune cell infiltration—particularly M1 macrophage polarization—was explored, and the interaction between CALCA and PNOC was analyzed by correlation analysis and protein-protein interaction networks. Experimental validation was performed using RT-qPCR in *H. pylori*-infected THP-1 macrophages and SH-SY5Y neuronal cells, along with loss- and gain-of-function experiments to investigate regulatory relationships.

**Results:**

Transcriptomic analysis of *H. pylori*-infected patients identified 683 differentially expressed genes enriched in immune and inflammatory pathways. Immune profiling further revealed prominent M1 macrophage polarization with increased γδT cells and B lymphocytes. Among the DEGs, four CGRP-related hub genes (PNOC, ICAM1, MMP9, and NFE2L1) were identified and found upregulated in *H. pylori*-infected macrophages. Correlation analyses showed PNOC, ICAM1, and MMP9 positively associated with M1 macrophages and their canonical markers, with knockdown of each gene attenuating M1 marker expression. In contrast, NFE2L1 showed no significant correlation, yet its knockdown increased M1 marker expression. Among the hub genes, only PNOC correlated positively with CALCA; both were upregulated in *H. pylori*-infected neuronal cells. Notably, PNOC knockdown in macrophages reduced CALCA expression in co-cultured neurons, while overexpression had the opposite effect, suggesting macrophage-derived PNOC may regulate neuronal CGRP expression.

**Conclusion:**

*H. pylori* infection may promote migraine and functional dyspepsia through gastric inflammation and neuroimmune signaling. This process involves four key genes—PNOC, ICAM1, MMP9, and NFE2L1—that regulate M1 macrophage polarization and pro-inflammatory responses. Among them, PNOC appears to link gastric inflammation to neurological symptoms by modulating neuronal CGRP. Together, these findings point to gut-brain axis involvement in this comorbidity and may inform future therapies targeting *H. pylori* eradication and CGRP-related pathways.

## 1 Introduction

Migraine is a complex neurological disorder characterized by recurrent, throbbing headaches often accompanied by nausea, vomiting, and extreme sensitivity to light and sound. Unlike typical headaches, migraines can last for hours or even days, significantly impacting daily life (1–3). According to the 2021 Global Burden of Disease Report, migraine affects approximately 28% of the global population and has emerged as the leading cause of disability among individuals aged 5–59 years with neurological disorders (4). A common comorbidity in migraine patients is functional dyspepsia, a condition characterized by a range of symptoms originating from the gastroduodenal region, such as nausea, vomiting, and heartburn. Notably, functional dyspepsia presents with normal endoscopic findings and no evidence of structural disease (5–7). The pathogenesis of functional dyspepsia involves multiple contributing factors, including gastrointestinal motility disorders, that *Helicobacter pylori (H. pylori)* infection, and visceral hypersensitivity (8–11). Emerging evidence suggests that in migraine patients, dysregulation of the autonomic nervous system and neurogenic inflammation may play critical roles as potential pathophysiological links between migraine and functional dyspepsia (12, 13). However, the exact molecular mechanisms connecting these two conditions remain incompletely understood and require further exploration.

In recent years, calcitonin gene-related peptide (CGRP) antagonists have emerged as migraine-specific preventive treatments, highlighting the pivotal role of CGRP in migraine pathogenesis (14–16). CGRP is a 37-amino acid neuropeptide encoded by the CGRP gene, existing in two isoforms generated through alternative mRNA splicing: α-CGRP and β-CGRP. While β-CGRP differs by only one amino acid and is primarily expressed in the brain, sensory ganglia, and thyroid gland, α-CGRP is predominantly found in the central and peripheral nervous systems as well as various endocrine tissues. Importantly, α-CGRP has been shown to mediate multiple physiological functions, including nociception, cardiovascular homeostasis, digestive regulation, and mineral metabolism (17). Growing evidence suggests that the gut-brain axis plays a critical role in linking gut microbiota to neurological disorders, including migraine (18). Within this context, CGRP has been implicated as a potential mediator in microbiota-neurological disorder interactions (19). However, the precise molecular mechanisms by which CGRP modulates the relationship between gut microbiota and neurological diseases remain unclear and warrant further investigation.

*H. pylori* is a Gram-negative spiral bacterium that typically inhabits the human stomach and duodenum, causing gastric mucosal inflammation (20). Previous studies have demonstrated a significantly higher prevalence of *H. pylori* infection in migraine patients (∼45%) compared to healthy controls (∼33%) (21). This association is further supported by a randomized, double-blind controlled trial, which demonstrated that *H. pylori* eradication therapy effectively reduced migraine-related disability compared to a placebo (22). Additionally, *H. pylori* eradication has been proven to alleviate symptoms of functional dyspepsia in infected patients (23). Together, these findings suggest a potential causal role of *H. pylori* in both migraine and functional dyspepsia (24, 25). During chronic colonization, *H. pylori* may penetrate the lamina propria, directly modulating the local immune milieu and interacting with sensory neurons—a process implicated in neurogenic inflammation (26, 27). However, the precise mechanisms by which *H. pylori* contributes to migraine pathogenesis or migraine-associated functional dyspepsia remain unclear.

*H. pylori* infection triggers gastric mucosal inflammation, with pro-inflammatory cytokines (e.g., IL-1β, TNF-α) recruiting immune cells, including macrophages, to the site of infection (28). Macrophages, central regulators of inflammatory responses, exhibit remarkable plasticity, polarizing into distinct phenotypes: the pro-inflammatory M1 state (induced by IFN-γ, TNF-α, LPS, et. al) to amplify inflammation through reactive oxygen species and nitric oxide production or the anti-inflammatory M2 state (driven by IL-4, IL-10, IL-13, et al.) to promote tissue repair and immune regulation (29–35). Notably, activated macrophages may sensitize meningeal afferents via Pannexin 1 signaling, potentially linking gastric inflammation to migraine pain (36, 37). Moreover, elevated macrophage activation in functional dyspepsia patients suggests a shared pathway between *H. pylori* infection and dyspeptic symptoms (38, 39). Despite evidence implicating macrophage polarization in *H. pylori* persistence and neuroinflammation, its role in migraine-associated functional dyspepsia remains unexplored.

This study therefore aims to systematically investigate the role of *H. pylori* infection, particularly through its induction of M1 macrophage polarization, in the comorbidity of migraine and functional dyspepsia. We will examine how *H. pylori* infection drives M1 polarization and subsequently modulates the expression of key neuroinflammatory mediators, including PNOC and CGRP, to establish functional gut-brain communication. By elucidating the dual impact of M1 polarization on both gastric inflammation and neural sensitization, our study seeks to uncover novel mechanisms underlying the migraine-associated functional dyspepsia, ultimately providing new insights for targeted therapeutic interventions.

## 2 Materials and methods

### 2.1 Dataset selection and CGRP-related genes identification

The *H. pylori* infection datasets used for the present study (GSE5081, GSE264263, GSE185270, GSE233973, GSE60427) were downloaded from NCBI Gene Expression Omnibus (GEO) database (https://www.ncbi.nlm.nih.gov/geo/). CGRP-related genes were collected from Gene Cards database (https://www.genecards.org/) and NCBI Gene database (https://www.ncbi.nlm.nih.gov/gene/).

### 2.2 Identification and analysis of differentially expressed genes

The *H. pylori* infection datasets were merged into one dataset collection for subsequent analysis, and principal component analysis (PCA) was used for the dataset collection. The R “sva” package was used to correct batch effects. The “limma” package was used for filtering and identifying differentially expressed genes (DEGs) based on the following cut-off criterion: |log2FC| > 1, *P* < 0.05. Volcano and heat maps were drawn using the obtained DEGs. Gene Ontology (GO) and Kyoto Encyclopedia of Genes and Genomes (KEGG) enrichment analyses were performed using the DAVID online tool (https://davidbioinformatics.nih.gov/). Gene Set Enrichment Analysis (GSEA) was performed on the relevant signal pathways of the dataset collection using GSEA software. The R “GSVA” and “GSEABase” packages were used for Gene Set Variation Analysis (GSVA) assessing the different biological functions between infected and normal samples with “h.all.v2023.2.Hs.symbols” of dataset collection.

### 2.3 Immune cell enrichment analysis

Gene expression data and LM22 files are evaluated for immune cell abundance in collections using the R CIBERSORT algorithm.

### 2.4 Machine learning analysis

By using the Least Absolute Shrinkage and Selection Operator (LASSO) regression method to filter the feature variables with the highest predictive ability, the model can be simplified, and its regularity, interpretability and prediction accuracy will be improved. The relevant variables were selected for model merging. The Support Vector Machine-Recursive Feature Elimination (SVM-REF) algorithm sorts the simple linear support vector machine established by SVM according to weight, eliminates the features with the lowest weight, and iteratively obtains the most predictive feature subset, significantly improving the prediction accuracy and feature selection efficiency of the model. The Random Forest (RF) method can have high levels of accuracy, specificity, and sensitivity without being limited by variable conditions, and is used to predict continuous variables without significant fluctuations. These LASSO, SVM-RFE, and RF machine learning analyses are implemented using R “glmnet”, “kernlab”, “e1071”, “caret”, and “random forest” packages. For RF, we selected the top 30 genes. The crossover genes in these analyses are considered to represent CGRP-related hub genes.

### 2.5 Construction and verification of the nomogram

The R “rms” package was utilized to establish a diagnostic nomogram for *H. pylori* infection risk while calibration plots and decision curve analysis (DCA) were performed using the “rmda” package in R.

### 2.6 Correlation analysis

The R “ggstatsplot” package was used to conduct correlation analysis between genes and between genes and immune cells.

### 2.7 Diagnostic value of biomarkers in *H. pylori* infection

A prediction model based on the expression of 4 hub genes was established to evaluate the predictive efficacy of the identified hub genes. A ROC curve was created to evaluate how well the biomarkers could differentiate between the healthy control (HC) and *H. pylori* samples (HP) using the “pROC” package in the R software. Subsequently, the AUC values were calculated to determine the trustworthiness of the identified biomarkers. Higher AUC values are indicative of superior predictive performance, with values approaching 1 suggesting heightened discriminatory ability between control and *H. pylori* samples. The validation cohorts GSE27411, GSE74492 and GSE92863 were similarly assessed by utilizing mRNA expression data from these datasets to construct ROC curves and compute AUC values. This was done in order to confirm the prognostic utility of the identified biomarkers across these independent datasets.

### 2.8 Consistency clustering analysis

The R “ConsensusClusterPlus” package was used to perform a consensus clustering based on the expression of central CGRP-related genes in order to determine the subgroups of *H. pylori* infected patients in the analysis dataset. This was done with the following settings: maxK = 9, clusterAlg = pam, distance = spearman.

### 2.9 Constructing PPI network through STRING

The STRING online database (https://string-db.org/) is used to construct Protein-Protein Interaction (PPI) networks between multiple molecules. All scores rank from 0 to 1, with 1 being the highest possible confidence.

### 2.10 Cell culture

THP-1 cells were maintained in RPMI-1640 medium (Servicebio) supplemented with 10% fetal bovine serum (FBS) at 37°C in a 5% CO_2_ humidified incubator, spread 3×10^5^ cells were seeded in a 24 well plate and allowed to adhere for 12 hours by adding PMA at a concentration of 200ng/mL. The medium was replaced and cells were allowed to recover for 24 hours. *H. pylori* was used to infect THP-1 cells at an MOI of 100 for 0, 3 and 6 hours. SH-SY5Y cells were maintained in RPMI-1640 medium supplemented with 10% fetal bovine serum at 37°C in a 5% CO_2_ humidified incubator. *H. pylori* was used to infect SH-SY5Y cells at an MOI of 100 for 0, 3, 6, and 12 hours. The cell lines present in this study were obtained from American Type Culture Collection (ATCC).

### 2.11 *H. pylori* culture

*H. pylori* strain PMSS1 was inoculated onto Brucella agar plates containing 10% sheep blood and cultured at 37°C in a microaerophilic environment (5% O_2_, 10% CO_2_, 85% N_2_) for 36 hours. Collect the culture to prepare a bacterial suspension, resuspend it in phosphate buffered saline (PBS), and adjust the bacterial concentration to 1×10^6^ colony forming units (CFU)/mL. During infection, the cultured *H. pylori* solution was diluted to the desired concentration using serum-free RPMI 1640 medium and co-cultured with THP-1 and SH-SY5Y cells.

### 2.12 Quantitative reverse transcription polymerase chain reaction

Total RNA was extracted from harvested cells using TRIzol reagent and reverse transcribed into cDNA using a Revert Aid First Strand cDNA Synthesis Kit (Thermo Fisher Scientific, United States). Quantitative real-time PCR was performed using SGExcel FastSYBR Mixture (Sangon Biotech, China) and 10 μM forward and reverse primers on a LightCycler instrument (Roche, Indianapolis, IN, United States). The primer sequences were listed. The relative expression of the mRNA was calculated using the 2^−ΔΔCt^ method and normalized to β-actin. The specific primers subjected to RT-qPCR analysis are listed as follows:

β-actin forward primer: 5’-AGTTGCGTTACACCCTTTCTTG-3’
β-actin reverse primer: 5’-CACCTTCACCGTTCCAGTTTT-3’
CALCA forward primer: 5’-GTCAAGGCACAGCATTACCA-3’
CALCA reverse primer: 5’-CCCTATTGACATTGGTGGCTCT-3’
PNOC forward primer: 5’-CCAGTGTGTTCAGCAGTTGTC-3’
PNOC reverse primer: 5’-GAAGCCCCCAAATCTCTTCTG-3’
CD80 forward primer: 5’- GGCCCGAGTACAAGAACCG-3’
CD80 reverse primer: 5’- TCGTATGTGCCCTCGTCAGAT-3’
ICAM1 forward primer: 5’- ATGCCCAGACATCTGTGTCC-3’
ICAM1 reverse primer: 5’- GGGGTCTCTATGCCCAACAA-3’
MMP9 forward primer: 5’- GGGACGCAGACATCGTCATC-3’
MMP9 reverse primer: 5’- TCGTCATCGTCGAAATGGGC-3’
NFE2L1 forward primer: 5’- CATTCTGCTGAGTTTGATTGGGG-3’
NFE2L1 reverse primer: 5’- TTGTGGAACTGGGTCTGAGTAT-3’

### 2.13 Small interfering RNA transfection and plasmid transfection for overexpression

For siRNA transfection, a mixture of 5µL siRNA and 5µL Lipofectamine 2000 reagent was combined with 500µL Opti-MEM for approximately 1.5×10^6^ cells. After 6 hours, the medium was replaced, and cells were collected after 3 days. For plasmid transfection, 2.5 µg of plasmid DNA was mixed with 5µL Lipofectamine 3000 reagent and 5µL P3000 and 250µL Opti-MEM for about 1.5×10^5^ cells, collecting cells after 2 days. siRNAs were sourced from Hippo Biotechnology Co., Ltd. Plasmids, including pEGFP and pEGFP-PNOC, were obtained from Shandong Jipeng Biotechnology Co., Ltd., Jinan, China.

### 2.14 Cell co-culture

Conditioned medium was harvested from THP-1 cells (previously subjected to siRNA knockdown or gene overexpression) following a 6-hour co-culture with *H. pylori*. After centrifugation (6000 rpm, 10 min), the supernatant was supplemented with 200 µL of serum and applied to SH-SY5Y cells. These cells were collected for analysis after 12 hours of incubation with *H. pylori*.

### 2.15 Statistical analysis

All the experiments were repeated in triplicates. All results are presented as mean ± SD. All statistical tests were conducted using R version 4.3.3. Pearson and Spearman correlation analyses were used to test the significance of the correlation. The data comparison was conducted using Student’s t-test or Wilcoxon test, with a significance threshold of *P* < 0.05. The WeiShengXin platform (https://www.bioinformatics.com.cn), SangerBox platform (http://www.SangerBox.com/) and R v4.3.3 were utilized for data visualization. Additionally, these tools were employed to analyze and interpret the results obtained from the statistical tests. * *P* < 0.05, ** *P* < 0.01, *** *P* < 0.001 and **** *P* < 0.0001 were considered as significant different. *P* ≥ 0.05 was considered not significant (–).

## 3 Results

### 3.1 Identification of DEGs in HC and *H. pylori* infected patients

**Figure 1:**
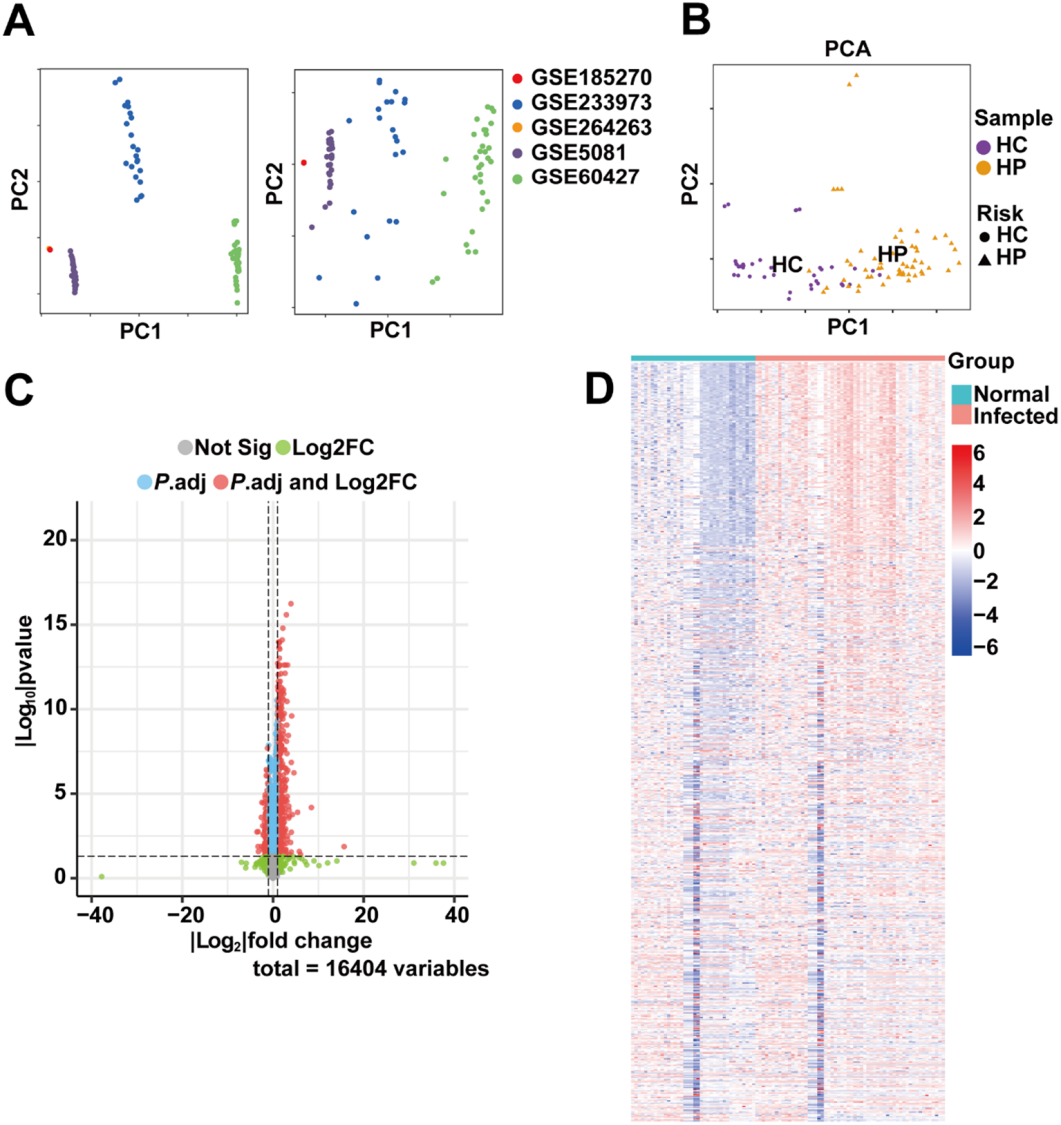
DEGs identification in HC and HP infected patients. **(A)** PCA graphs demonstrating distinct clustering patterns of five datasets before and after batch correction. **(B)** PCA diagram demonstrating distinct clustering patterns between healthy controls (HC, n = 38) and *H. pylori*-infected patients (HP, n = 58). **(C)** Volcano plot of DEGs between HC and HP groups (|log2FC| > 1, *P* < 0.05). The plot identifies 504 significantly upregulated (red) and 179 downregulated (blue) genes, with the most statistically significant DEGs positioned higher on the y-axis. **(D)** Hierarchically clustered heatmap of all significant DEGs, revealing consistent expression patterns within each group and clear separation between HC and HP samples. Rows represent genes while columns represent individual samples.

It was reported that *H. pylori* infection exhibits higher prevalence among migraine patients, suggesting its potential involvement in migraine pathogenesis (21). To further elucidate the underlying molecular mechanisms, we conducted comparative transcriptomic analysis using *H. pylori* infection datasets. To ensure robust and generalizable findings, we compiled a dataset collection comprising five *H. pylori*-related gene expression datasets (GSE5081, GSE264263, GSE185270, GSE233973, and GSE60427). This integrated dataset included 38 healthy controls (HC) and 58 *H. pylori*-infected patients (HP). As shown in **Figure 1A**, PCA graphs were generated to demonstrate the existence of batch effects and the effectiveness of the correction. The corrected data exhibited strong intra-group consistency in gene expression profiles, while revealing significant differences between HC and HP groups (**Figure 1B**), confirming the suitability of the dataset for downstream analysis. Differential expression analysis identified 683 significantly altered genes (*P* value < 0.05, |log_2_ (FC)| > 1), including 504 upregulated and 179 downregulated genes in HP compared to HC. These DEGs were visualized using a volcano plot (**Figure 1C**) and a heatmap (**Figure 1D**), highlighting distinct expression patterns between the two groups.

### 3.2 Functional enrichment analysis reveals immune-inflammatory pathways in *H. pylori* infection

**Figure 2:**
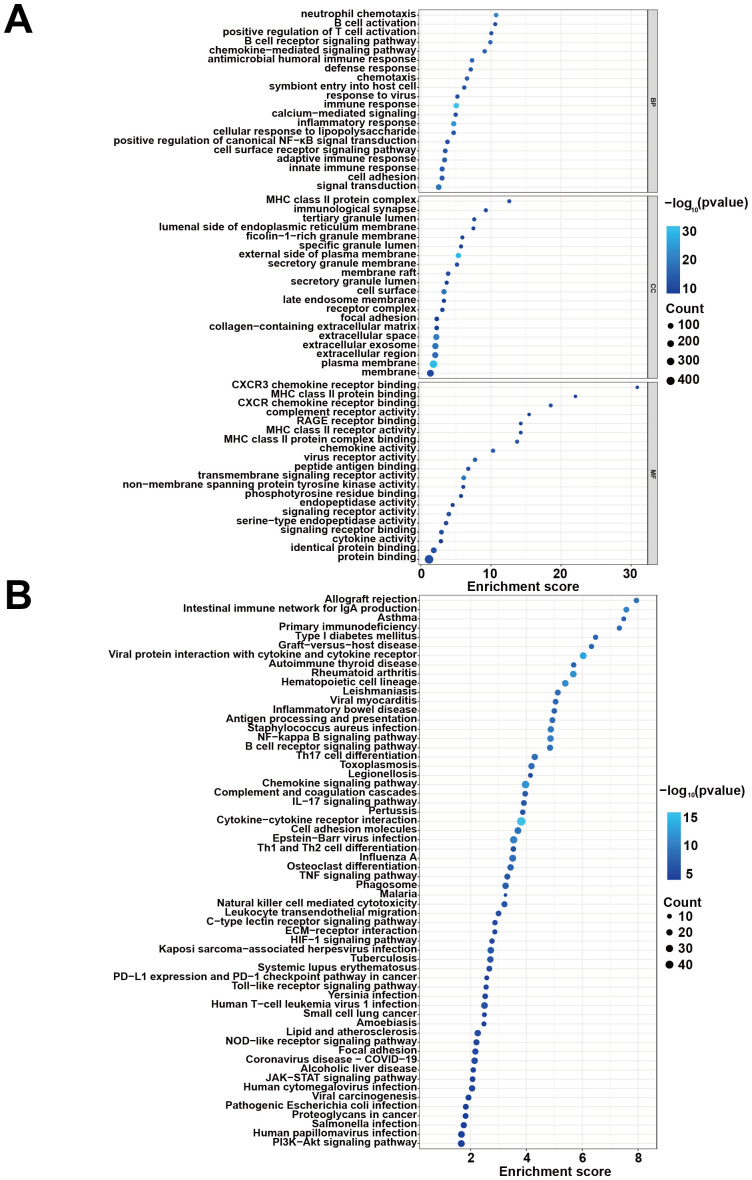
DEGs functional enrichment analysis. **(A)** GO term enrichment across biological processes, cellular components, and molecular functions. **(B)** KEGG pathway network showing key immune and inflammatory pathways.

To elucidate the potential molecular mechanisms by which *H. pylori* infection may contribute to migraine pathogenesis, we performed comprehensive functional enrichment analysis of the identified DEGs using DAVID. In the GO analysis, the DEGs were significantly enriched in several key biological processes, including neutrophil chemotaxis, immune response, and inflammatory response. For cellular components, the DEGs were predominantly localized in the MHC class II protein complex, immunological synapse, and tertiary granule lumen. Regarding molecular functions, the DEGs were notably associated with C-X-C chemokine receptor type 3 (CXCR3) chemokine receptor binding, MHC class II protein binding, and CXCR chemokine receptor binding (**Figure 2A**). KEGG pathway analysis further revealed that the DEGs were closely linked to critical signaling pathways, such as cytokine-cytokine receptor interaction, intestinal immune network for IgA production, inflammatory bowel disease, Th17 cell differentiation, IL-17 signaling pathway, and Th1/Th2 cell differentiation (**Figure 2B**). These enrichment results suggest that the identified DEGs may play a role in the sustained colonization of *H. pylori* and chronic persistent inflammation, which could in turn have implications for the development of migraine and functional dyspepsia. Further investigation will be helpful to clarify the precise mechanisms linking these pathways to the clinical manifestations.

### 3.3 Enhanced inflammatory and immune responses following *H. pylori* infection

**Figure 3:**
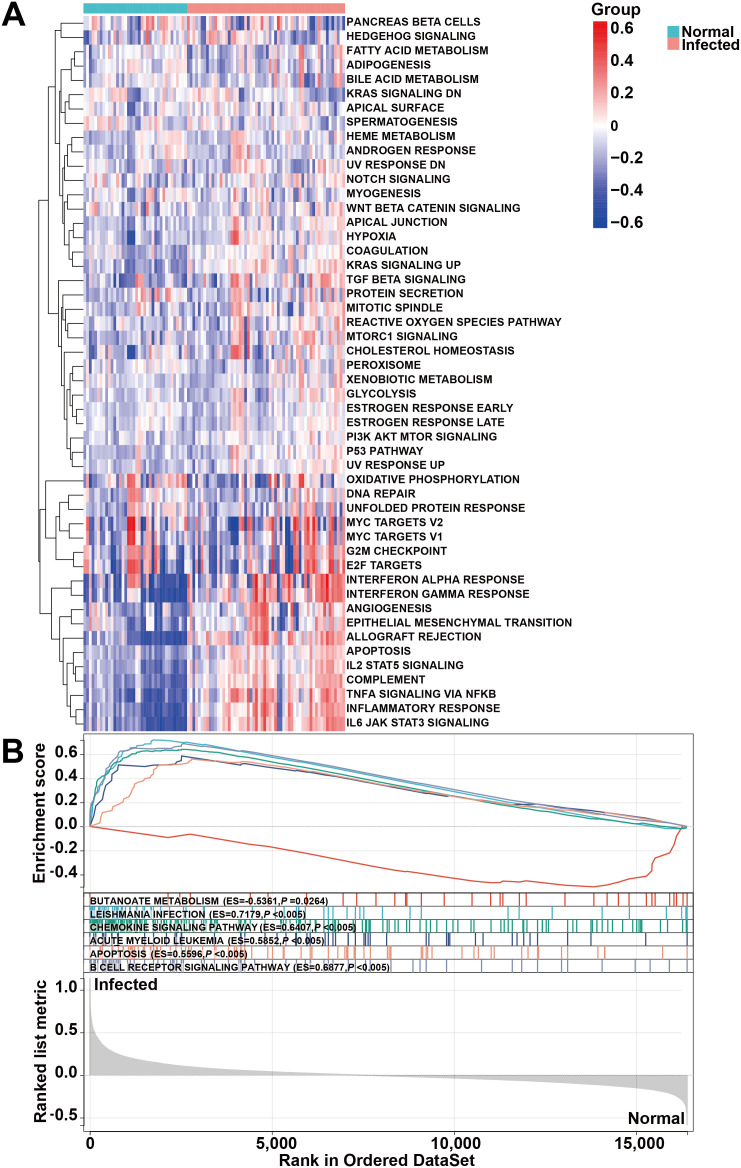
Pathway enrichment analyses comparing HC and *H. pylori*-infected patients. **(A)** GSVA enrichment results of HALLMARK pathway. **(B)** GSEA enrichment results of KEGG pathway.

To further investigate the impact of *H. pylori* infection on migraine-related pathways, we conducted Gene Set Variation Analysis (GSVA) to assess pathway activity alterations. The heatmap analysis revealed significant upregulation of key inflammatory and immune-related pathways, including the IL6-JAK-STAT3 signaling pathway, inflammatory response, and IL2-STAT5 signaling pathway, following *H. pylori* infection (**Figure 3A**). GSVA enrichment results demonstrated that *H. pylori* infection not only amplifies inflammatory responses but also triggers cell apoptosis and modulates T-cell immune function and metabolic processes. Further validation through GSEA identified additional dysregulated pathways, such as the B cell receptor signaling pathway, T cell receptor signaling pathway, and apoptosis pathway (**Figure 3B**). These analyses collectively revealed three major consequences of *H. pylori* infection: (1) establishment of a sustained inflammatory microenvironment, (2) functional dysregulation of immune cells, and (3) increased apoptotic activity. Collectively, these findings suggest that *H. pylori* infection potentiates neuroinflammation and promotes immune cell activation, which may contribute to migraine pathogenesis through chronic inflammatory and immune-mediated mechanisms.

### 3.4 M1 macrophages mediate the exacerbation of inflammatory response and activation of immune cells

**Figure 4:**
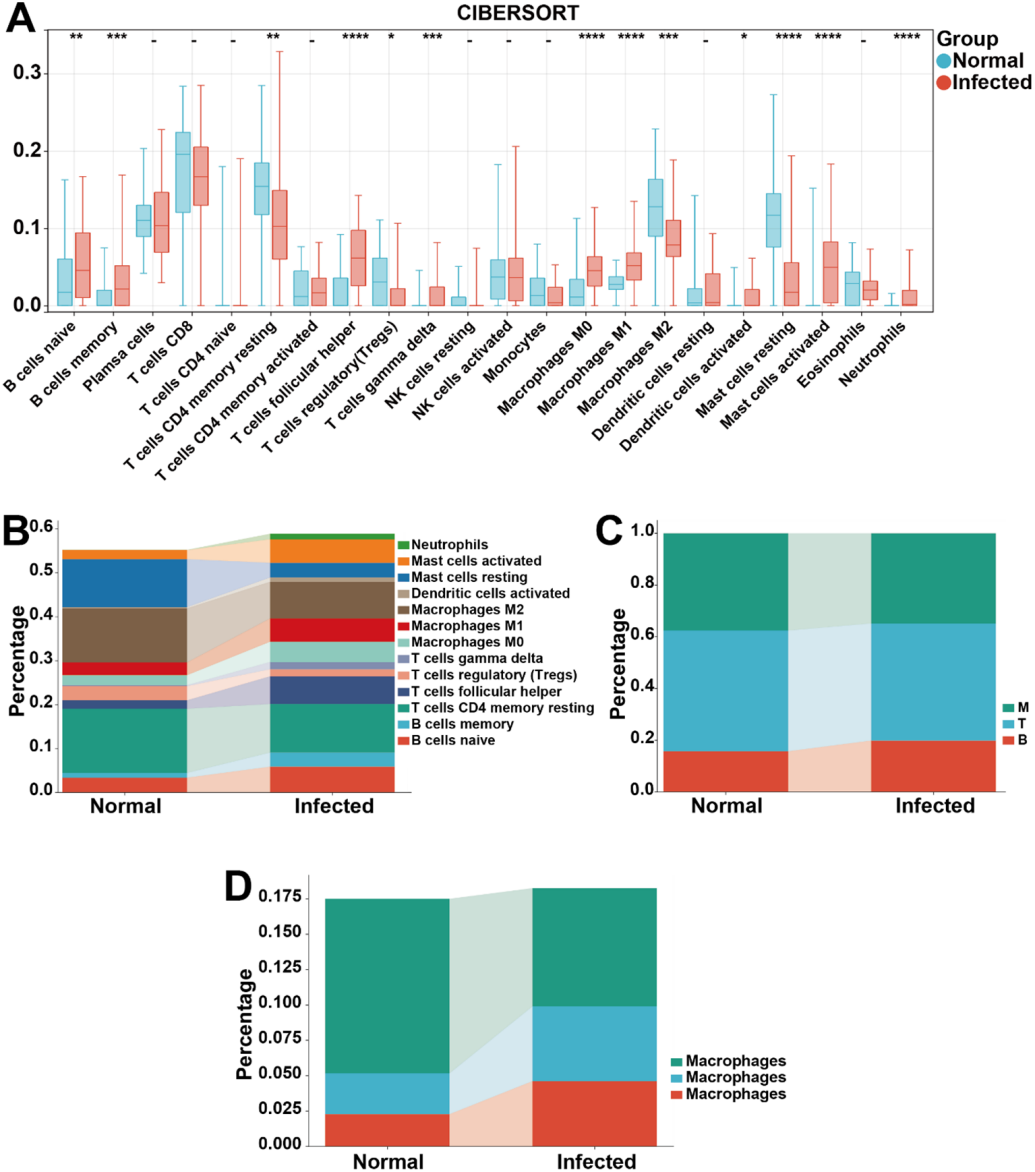
Immune cell profiling in *H. pylori*-infected patients vs. HCs. **(A)** Comparative analysis of 22 immune cell subtypes between *H. pylori*-infected patients and HCs. **(B)** Differential infiltration levels of significantly altered immune cell populations. **(C)** Proportional distribution of major immune cell lineages: myeloid cells (M), T cells (T), and B cells **(B)**. **(D)** Relative abundance of macrophage subtypes (M0, M1, M2) within the myeloid compartment. *P < 0.05, **P < 0.01, ***P < 0.001, ****P < 0.0001, -, not significant.

Immune cells are recognized as key mediators in chronic persistent inflammation (40–43), and emerging evidence underscores their critical involvement in migraine pathogenesis (44–46). To elucidate the impact of *H. pylori* on immune cell activation, we analyzed the immune microenvironment in our dataset. Significant alterations were observed across multiple immune components, including naive B cells, memory B cells, follicular helper T cells (Tfh), gamma delta T (γδT) cells, M0/M1/M2 macrophages, activated dendritic cells, activated/resting mast cells, neutrophils, CD4^+^ memory/resting T cells, and regulatory T cells (Tregs) (**Figure 4A**). Further comparative analysis revealed pronounced immune disparities: Tfh, activated mast cells, and M1 macrophages exhibited the most marked increases in abundance, whereas resting mast cells, M2 macrophages, and CD4^+^ memory resting T cells showed the most significant reductions (**Figure 4B**). To systematically evaluate these changes, the 22 immune cell types were classified into three major categories: myeloid cells (M), T cells (T), and B cells (B). Strikingly, B cell infiltration levels were significantly elevated, while myeloid cell infiltration was markedly suppressed (**Figure 4C**). Among myeloid subsets, macrophages displayed the most dynamic shifts, with M1 polarization demonstrating the largest fold change in infiltration levels (**Figure 4D**). These findings collectively suggest that M1 macrophage polarization may contribute to inflammatory processes and T cell activation in the context of *H. pylori* infection, potentially serving as a possible mechanistic link in migraine development, thus providing valuable insights for future research on macrophages.

### 3.5 Identification and analysis of CGRP-related hub genes in migraine pathogenesis

**Figure 5:**
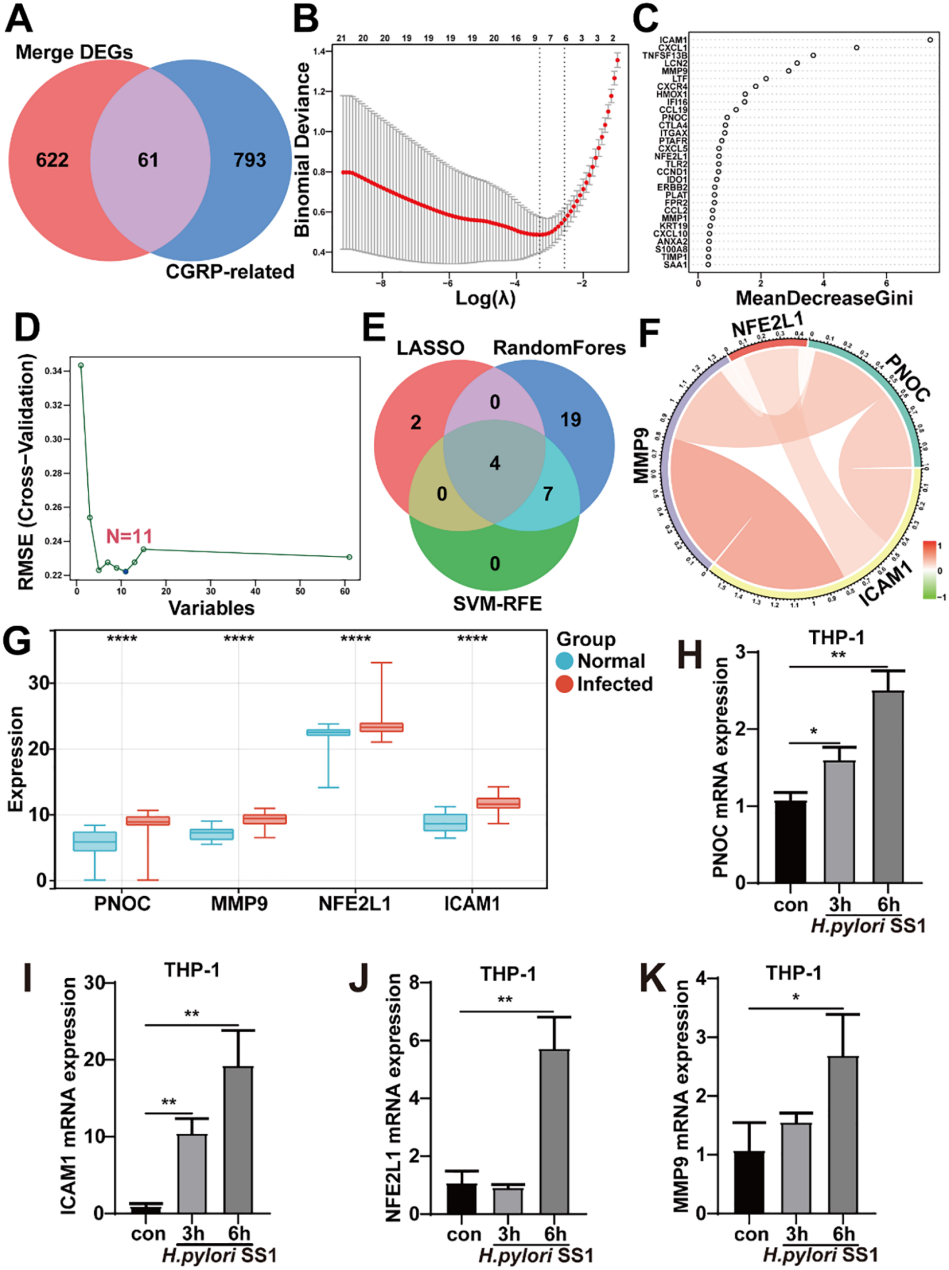
Machine learning-based identification of a CGRP-related gene signature in *H. pylori*-associated migraine. **(A)** Venn diagram showing the intersection between 683 *H. pylori*-related DEGs and 854 known CGRP-related genes, yielding 61 candidate genes. **(B-D)** Three machine learning approaches were employed to refine the signature: **(B)** LASSO regression with 10-fold cross-validation selected optimal predictive features; **(C)** RF analysis ranked the top 30 genes by importance score; and **(D)** SVM-RFE algorithm identified 11 key variables. **(E)** A Venn diagram highlighting overlap among candidate genes identified with these three machine learning algorithms. **(F)** A Circos plot displaying the relationship between the overlapping CGRP-related genes. **(G)** Differential expression of hub genes between HC and HP groups in the merged and batch-corrected dataset collection (GSE5081, GSE264263, GSE185270, GSE233973, GSE60427). **(H-K)** Verification of elevated hub gene expression in THP-1 cells after *H. pylori* infection. Data are shown as mean ± SD of n = 3 biological replicates, *P < 0.05, **P < 0.01, ***P < 0.001, ****P < 0.0001, two-tailed unpaired Student’s t test **(H-K)**.

CGRP has been established as a key mediator in migraine pathophysiology, with growing evidence indicating its regulation by gut microbiota (25). To investigate the potential association between *H. pylori* infection and CGRP signaling, we performed an intersection analysis of 683 *H. pylori*-related DEGs with 854 known CGRP-associated genes, identifying 61 CGRP-related DEGs that included important inflammatory and pain-regulating factors such as CXCR4, tumor necrosis factor (TNF), Matrix Metallopeptidase 9 (MMP9), and intercellular cell adhesion molecule-1 (ICAM1) (**Figure 5A**). Through a comprehensive machine learning approach combining LASSO regression (with generalized cross-validation), Random Forest analysis (selecting the top 30 most important genes), and SVM-RFE algorithm (identifying 11 optimal variables), we identified four core hub genes (**Figures 5B–E**): PNOC (encoding pain-regulating neuropeptides) (47), MMP9 (involved in inflammatory tissue remodeling) (48), ICAM1 (essential for leukocyte adhesion and inflammatory responses) (49) and nuclear factor erythroid 2 like bZIP transcription factor 1 (NFE2L1) (a negative regulator of inflammation) (50). Strikingly, the four hub genes exhibited strong inter-gene correlations (**Figure 5F**), suggesting their coordinated involvement in biological processes. Notably, all four genes were significantly upregulated following *H. pylori* infection (**Figure 5G**), a finding that was further validated in THP-1 cells (**Figures 5H–K**). These results collectively implicate them in *H. pylori*-associated migraine pathogenesis.

### 3.6 Clinical validation of the hub gene signature for *H. pylori* infection diagnosis

**Figure 6:**
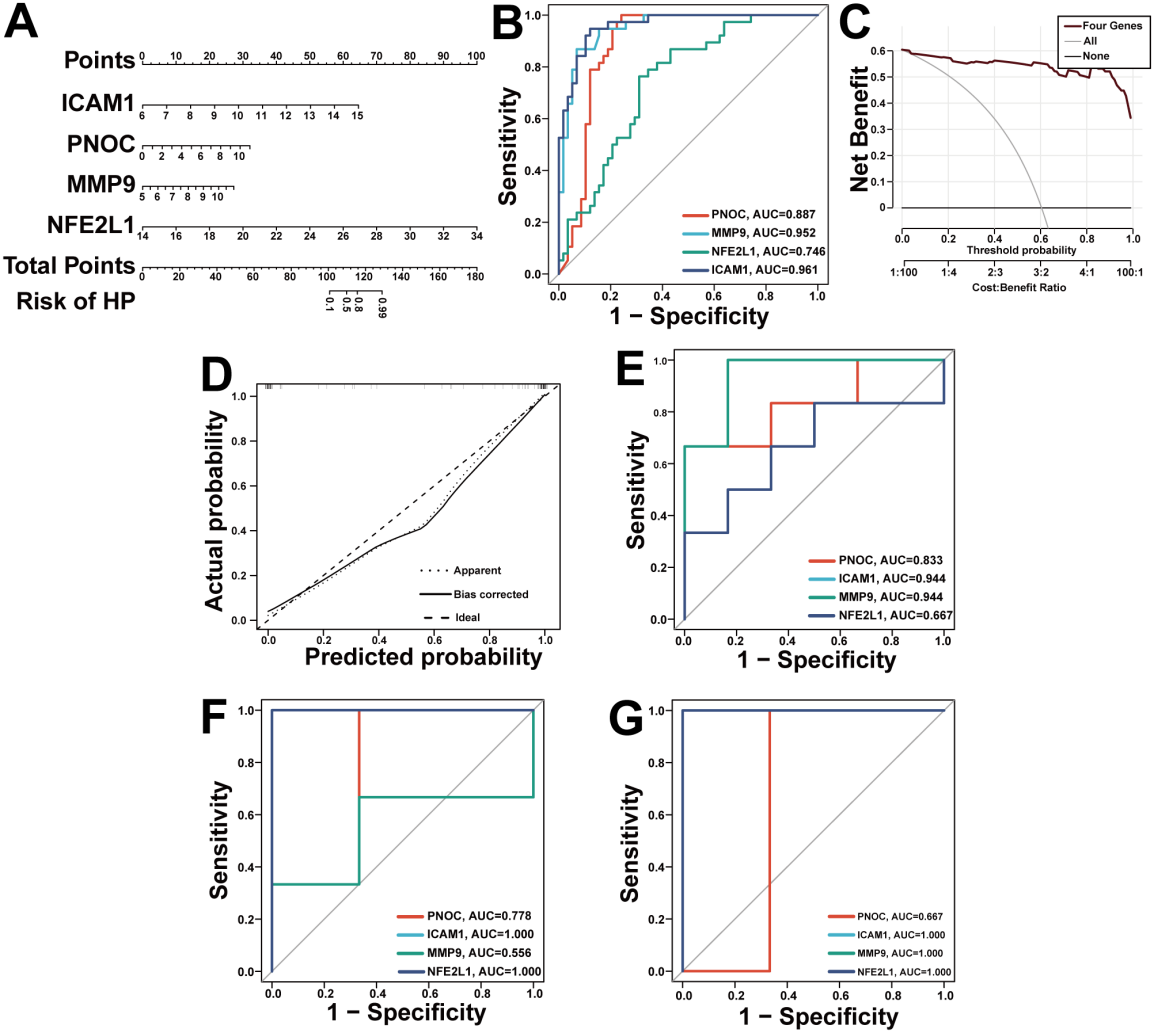
Clinical validation of the CGRP-related gene signature for *H. pylori*-associated migraine diagnosis. **(A)** A predictive nomogram integrating the four hub genes for clinical risk assessment. **(B)** ROC curve of the CGRP-related signature when used for the diagnosis of *H. pylori*. **(C)** Decision curve analysis for the established nomogram. **(D)** Calibration plot assessing the robustness of nomogram predictions. **(E-G)** Validate and test the predictive ability of predictive models on **(E)** GSE27411, **(F)** GSE74492, **(G)** GSE92863.

To explore the potential clinical applicability of these molecular findings, we developed a diagnostic nomogram incorporating the four hub genes (PNOC, ICAM1, MMP9, and NFE2L1) to predict *H. pylori* infection status (**Figure 6A**). Preliminary evaluation of the model’s discriminative ability revealed an AUC of 0.92 (95% CI: 0.87–0.96; **Figure 6B**), suggesting that the four-gene signature may have potential in distinguishing infected patients from healthy controls. Decision curve analysis indicated a positive net clinical benefit across threshold probabilities from 0.06 to 1.0 (**Figure 6C**), raising the possibility that the nomogram might offer some value in informing clinical decisions. Calibration curves demonstrated generally acceptable agreement between predicted and observed outcomes (**Figure 6D**), though some deviations were noted at extreme probability ranges. External validation in three independent cohorts yielded AUC values of 0.90, 0.88, and 0.91, respectively (**Figures 6E–G**), providing preliminary evidence for the potential generalizability of this signature. Collectively, these observations suggest that the four hub genes may hold promise as candidate biomarkers for *H. pylori* infection, although additional studies in larger and more diverse populations will be important to further evaluate their diagnostic utility.

### 3.7 Consistency clustering validates hub gene expression patterns and immune infiltration characteristics

**Figure 7:**
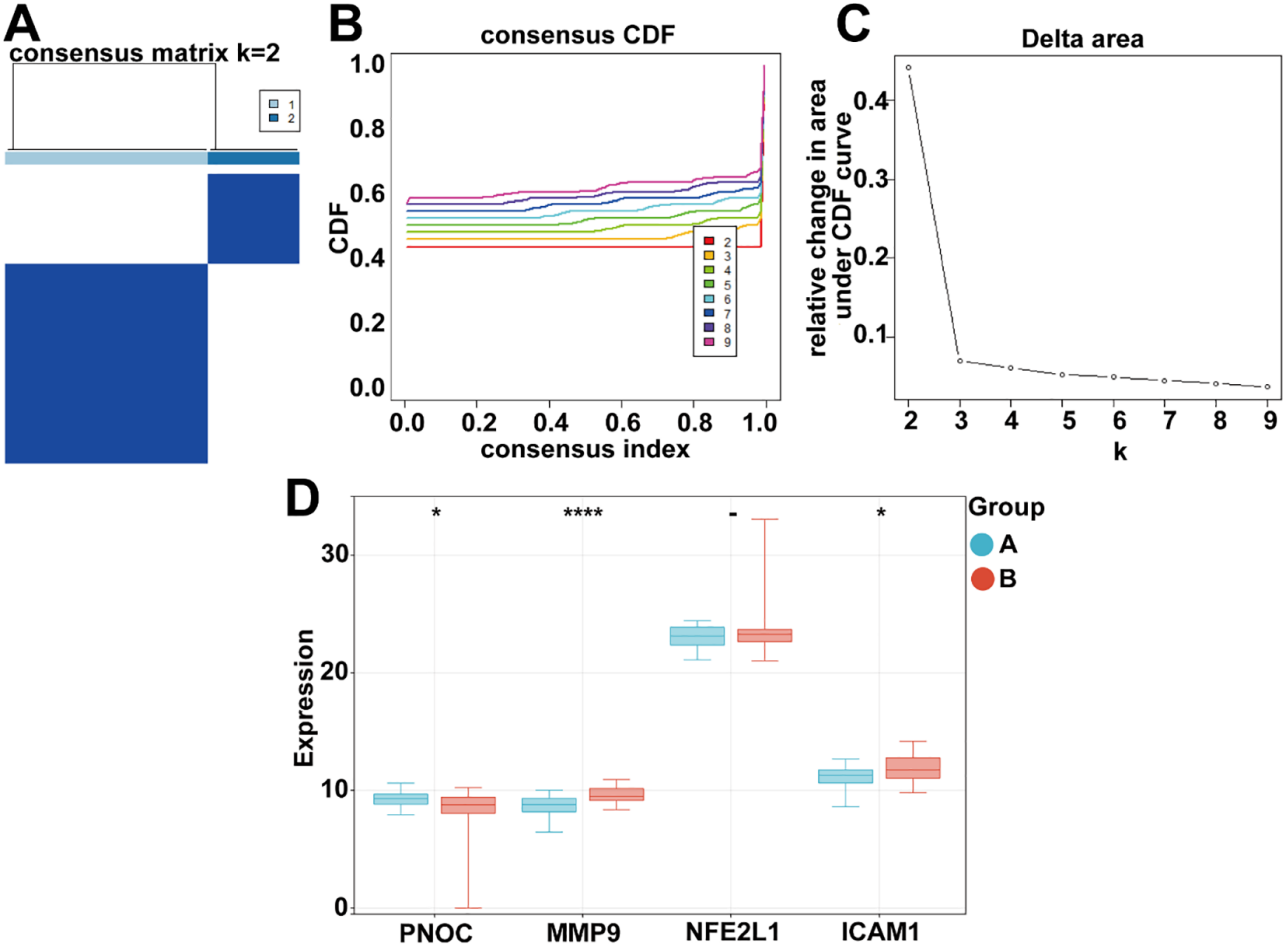
Identification of CGRP-related molecular subtypes in *H. pylori*-associated migraine. **(A)** Unsupervised consensus clustering of the merged dataset based on DEGs revealed distinct molecular patterns. **(B)** The consistency index analysis (y-axis) versus cumulative distribution function (CDF, x-axis) demonstrated optimal clustering stability. **(C)** The delta area plot (change in CDF area under curve) confirmed k = 2 as the most appropriate number of clusters. **(D)** Comparative analysis of hub gene expression between the two identified subtypes showed significant upregulation of PNOC, MMP9, and ICAM1 in the *H. pylori*-enriched subtype, while NFE2L1 showed comparable expression levels between groups. *P < 0.05, ****P < 0.0001, -, not significant.

To further explore the possible involvement of CGRP-related hub genes in *H. pylori*-associated migraine, we performed consistency clustering analysis. The optimal clustering solution was determined at k = 2, which stratified all samples into two distinct molecular subtypes (Cluster A and Cluster B), as illustrated by the consensus matrix (**Figure 7A**), cumulative distribution function (CDF) plot (**Figure 7B**), and relative change in area under CDF curve (**Figure 7C**). Comparative analysis of hub gene expression between these two subtypes revealed notable differences in three of the four hub genes: PNOC (*P* = 0.003, fold change = 2.1), MMP9 (*P* = 0.011, fold change = 1.8), and ICAM1 (*P* = 0.007, fold change = 1.9) (**Figure 7D**). Notably, these three genes showed coordinated overexpression in the cluster associated with predominant *H. pylori* infection status (Cluster B). In contrast, NFE2L1 expression levels remained comparable between the two subtypes (*P >*0.05), suggesting its potential role as a baseline regulator rather than a differentially expressed marker in this context. The observed separation of samples into biologically distinct clusters, coupled with the consistent gene expression differences, offers some support for the possibility that *H. pylori* infection could influence CGRP pathway activity through these hub genes, potentially contributing to migraine-related pathophysiology. Further investigation will be helpful to clarify the precise mechanisms underlying these associations.

### 3.8 Hub gene may be involved in macrophage polarization

**Figure 8:**
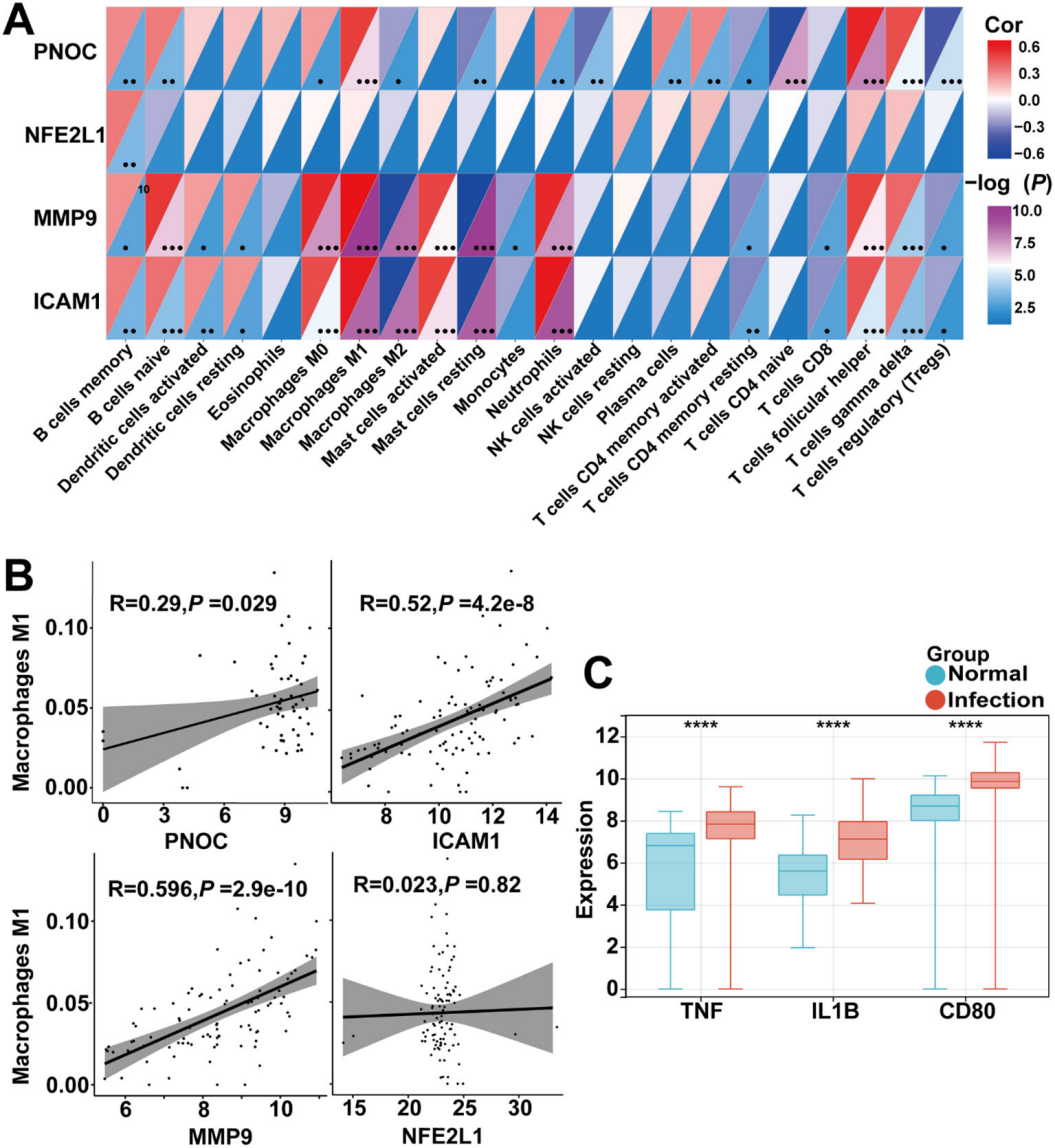
Correlation analysis and experimental verification of immune cells and cell markers. **(A)** Immune cell correlation profile of hub genes across 22 immune cell types. **(B)** Hub genes expression correlations with M1-polarized macrophage signatures. **(C)** Comparative expression profiling of key inflammatory cytokines between *H. pylori*-infected and control groups. *P < 0.05, **P < 0.01, ***P < 0.001, ****P < 0.0001, -, not significant.

Mounting evidence has firmly established that *H. pylori* infection promotes M1-polarization of macrophages, potentially contributing to persistent inflammation (51, 52). To analyze the involvement of PNOC, ICAM1, MMP9, and NFE2L1 in immune regulation, we explored their possible relationships with immune cell subsets. Correlation analysis revealed that PNOC, ICAM1, and MMP9 exhibited significant positive correlations with M1 macrophages, neutrophils, and γδT cells, whereas NFE2L1 showed no significant association with immune cells (**Figure 8A**). It has been reported that M1 macrophages may play a role in neutrophil activation and γδT cell responses (53–55). These findings suggest that PNOC, ICAM1, and MMP9 may participate in pro-inflammatory responses, while NFE2L1 likely plays a distinct regulatory role in the immune microenvironment of *H. pylori* infection. Further quantitative correlation analysis revealed positive associations between PNOC, ICAM1, MMP9 and M1 macrophage polarization, in contrast to NFE2L1 which showed no significant correlation (**Figure 8B**).

**Figure 9:**
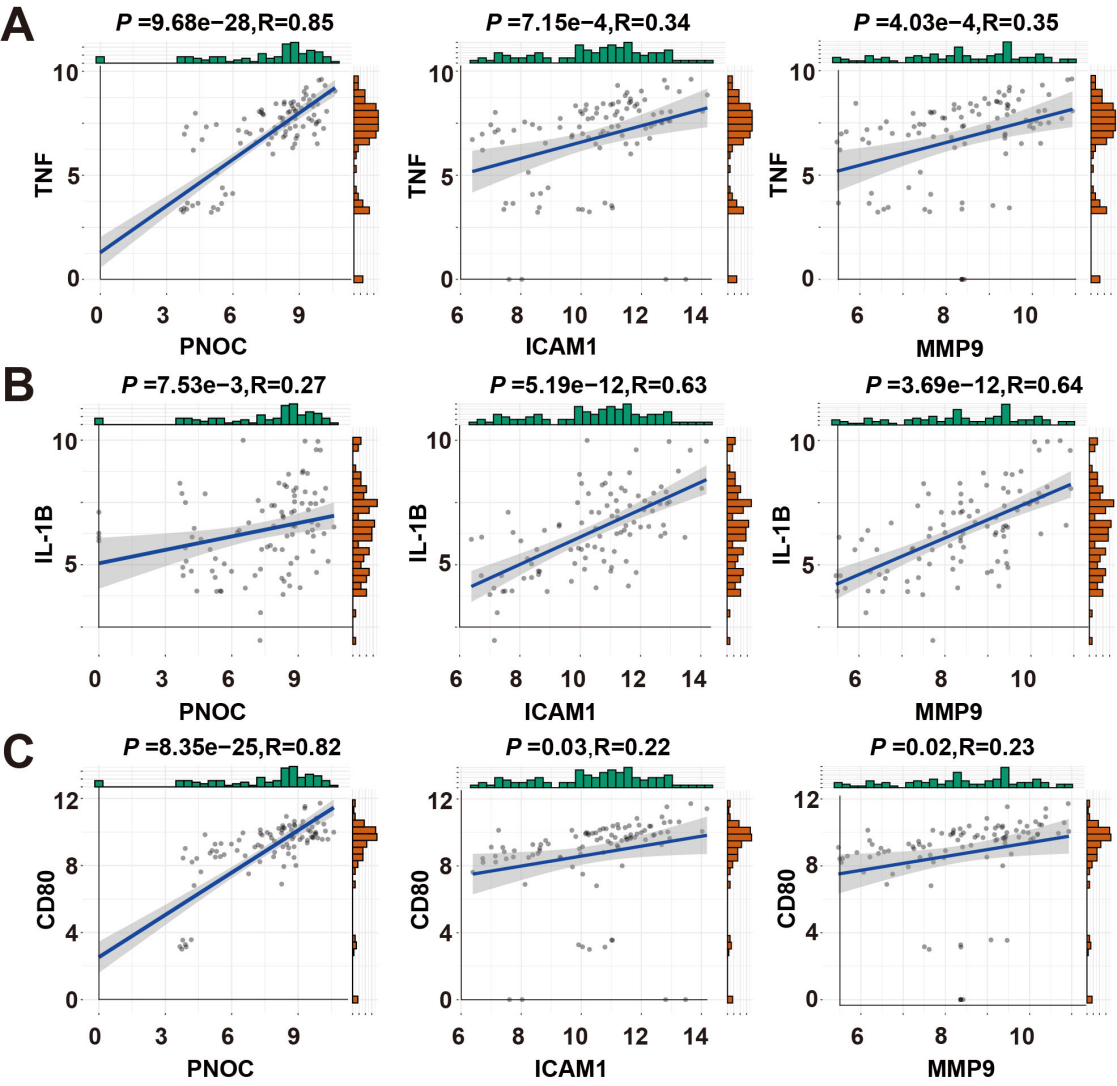
Correlation analysis of hub genes and cell markers. **(A)** Correlation analysis between hub genes and TNF. **(B)** Correlation analysis between hub genes and IL-1B. **(C)** Correlation analysis between hub genes and CD80.

To further explore the potential role of inflammatory cell infiltration and cytokine secretion, we analyzed three key inflammatory biomarkers: TNF-α, IL-1β, and CD80 (classical M1 macrophage markers) (51, 53, 54). All three markers were significantly upregulated following *H. pylori* infection, underscoring the critical involvement of macrophages in chronic inflammation (**Figure 8C**). To investigate the relationship between PNOC, ICAM1, MMP9, NFE2L1 expression and M1 macrophage polarization, we analyzed their correlation with established M1 markers (TNF-α, IL-1β and CD80). Positive correlations were observed for PNOC, ICAM1 and MMP9 (**Figures 9A–C**, **Supplementary Figure 1**), indicating their potential involvement in M1 macrophage-mediated inflammatory responses.

**Figure 10:**
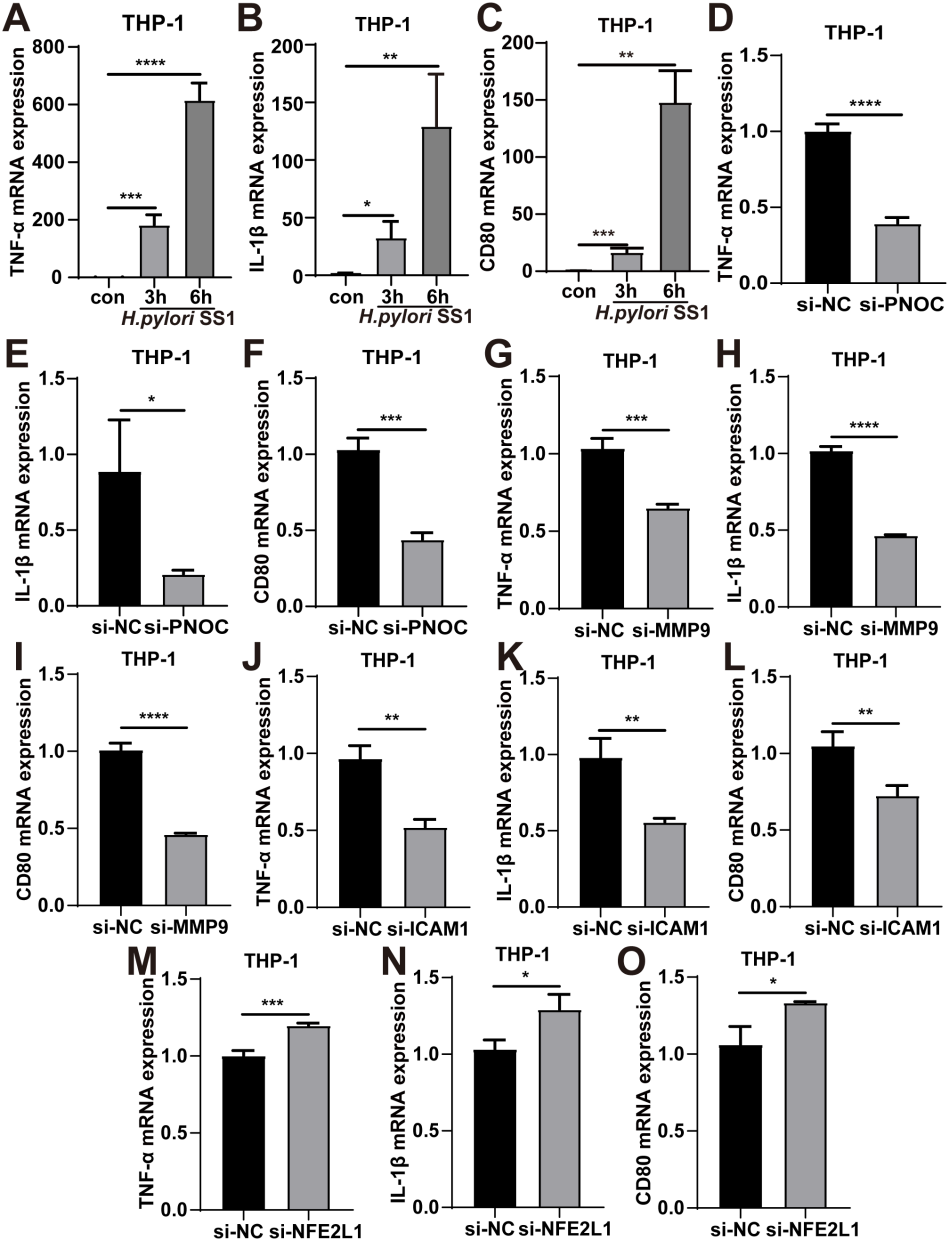
Functional validation of hub genes on macrophage M1 polarization by knockdown. **(A-C)** RT-qPCR verification of M1 polarization marker (TNF-α, IL-1β and CD80) expression in THP-1 cells after *H. pylori* infection. **(D-F)** RT-qPCR verification of M1 polarization marker (TNF-α, IL-1β and CD80) expression in THP-1 cells after knockdown PNOC. **(G-I)** RT-qPCR verification of M1 polarization marker (TNF-α, IL-1β and CD80) expression in THP-1 cells after knockdown MMP9. **(J-L)** RT-qPCR verification of M1 polarization marker (TNF-α, IL-1β and CD80) expression in THP-1 cells after knockdown ICAM1. **(M-O)** RT-qPCR verification of M1 polarization marker (TNF-α, IL-1β and CD80) expression in THP-1 cells after knockdown NFE2L1. All data are shown as mean ± SD of n = 3 biological replicates, *P < 0.05, **P < 0.01, ***P < 0.001, ****P < 0.0001, two-tailed unpaired Student’s t test.

RT-qPCR analysis showed increased TNF-α, IL-1β and CD80 expression after *H. pylori* infection (**Figures 10A–C**), consistent with M1 polarization. To explore the potential role of hub genes in this process, we knocked down each gene in THP-1 cells using siRNA. Knockdown of PNOC, ICAM1, or MMP9 was associated with reduced levels of M1 polarization markers (**Figures 10D–L**). In contrast, NFE2L1 knockdown led to increased expression of these markers (**Figures 10M–O**). Although NFE2L1 did not show significant correlations with immune cell populations in our bioinformatics analysis, this experimental observation is consistent with previous studies reporting its anti-inflammatory role (50). These findings raise the possibility that PNOC, ICAM1, and MMP9 may contribute to M1-associated inflammatory responses, while NFE2L1 might play a role in limiting such responses, potentially influencing chronic inflammation and bacterial persistence.

### 3.9 Validation of relationship between PNOC and CALCA in *H. pylori* infection

**Figure 11:**
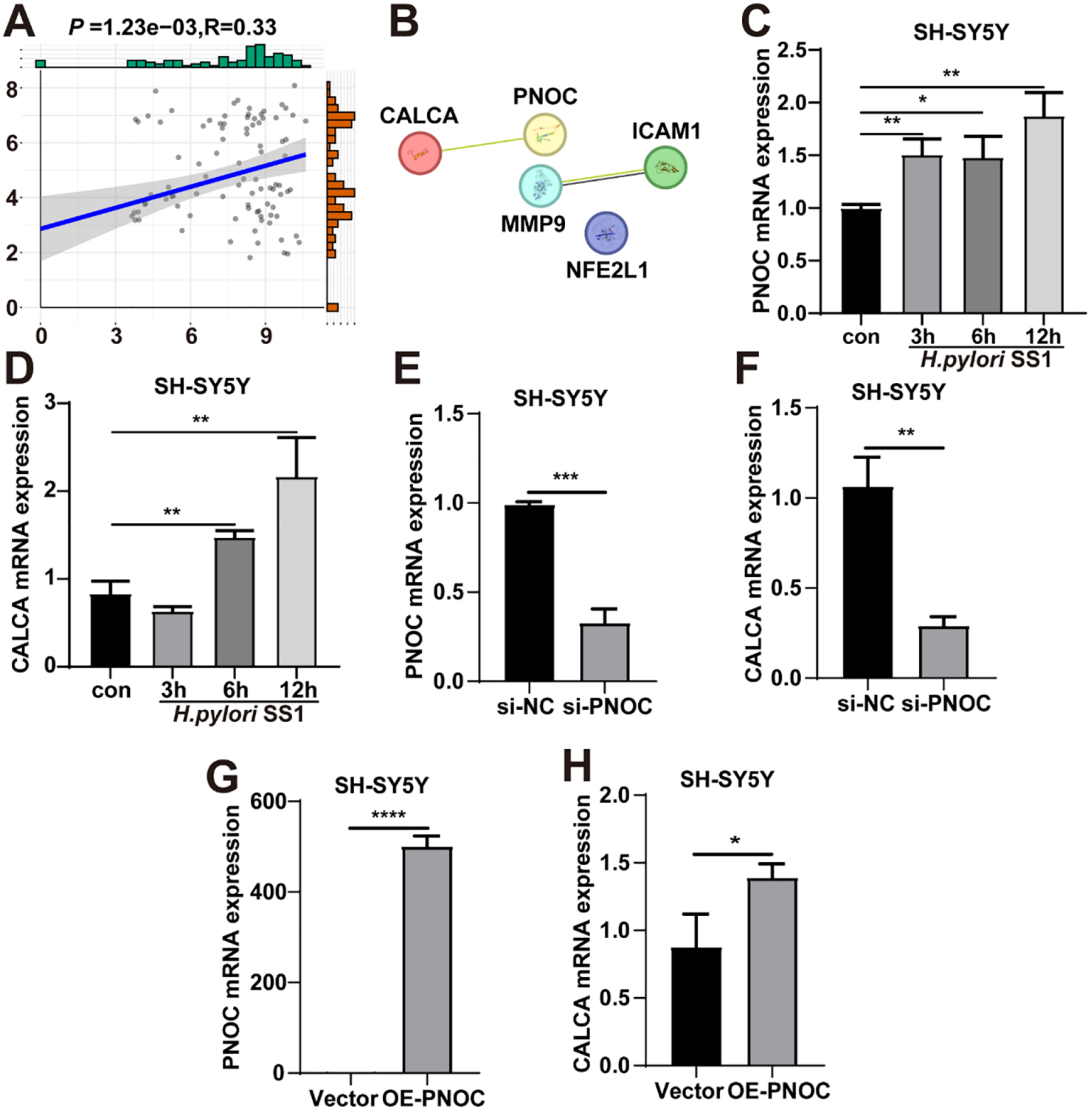
Experimental validation of the relationship between PNOC and CALCA in *H. pylori*-infected SH-SY5Y cells. **(A)** Correlation analysis between PNOC and CALCA. **(B)** PPI network showing the predicted interaction between CALCA and hub genes (STRING score between CALCA and PNOC: 0.526). **(C, D)** Relative mRNA expression levels of PNOC **(C)** and CALCA **(D)** in SH-SY5Y cells following *H. pylori* infection, as determined by RT-qPCR. **(E, F)** Expression of PNOC **(E)** and CALCA **(F)** in SH-SY5Y cells after siRNA-mediated knockdown of PNOC. **(G, H)** Expression of PNOC **(G)** and CALCA **(H)** in SH-SY5Y cells following PNOC overexpression. Data are shown as mean ± SD of n = 3 biological replicates, *P < 0.05, **P < 0.01, ***P < 0.001, ****P < 0.0001, two-tailed unpaired Student’s t test **(C–H)**.

To explore the relationship between the four hub genes (PNOC, ICAM1, MMP9, and NFE2L1) and CALCA, we performed correlation analysis. The results showed that only PNOC exhibited a significant positive correlation with CALCA (**Figure 11A**, **Supplementary Figure 2**). This finding was further supported by PPI network analysis using STRING, which revealed a moderate interaction score of 0.526 between CALCA and PNOC (**Figure 11B**). To validate these bioinformatics predictions, we examined the expression of PNOC and CALCA in *H. pylori*-infected cells. Due to the low basal expression of CALCA in THP-1 macrophages, we used SH-SY5Y neuroblastoma cells for this analysis. Following *H. pylori* co-culture, both genes appeared to be upregulated (**Figures 11C, D**), consistent with the possibility that they may play a role in *H. pylori*-associated migraine and functional dyspepsia. To further investigate the regulatory relationship between PNOC and CALCA, we knocked down or overexpressed PNOC in SH-SY5Y cells and examined subsequent changes in CALCA mRNA levels. RT-qPCR results showed that knockdown of PNOC was associated with reduced CALCA mRNA levels (**Figures 11E, F**), while overexpression of PNOC led to increased CALCA expression (**Figures 11G, H**). These findings raise the possibility that PNOC may positively regulate CALCA in neuronal cells, potentially contributing to the molecular mechanisms linking *H. pylori* infection to migraine and related gastrointestinal symptoms.

**Figure 12:**
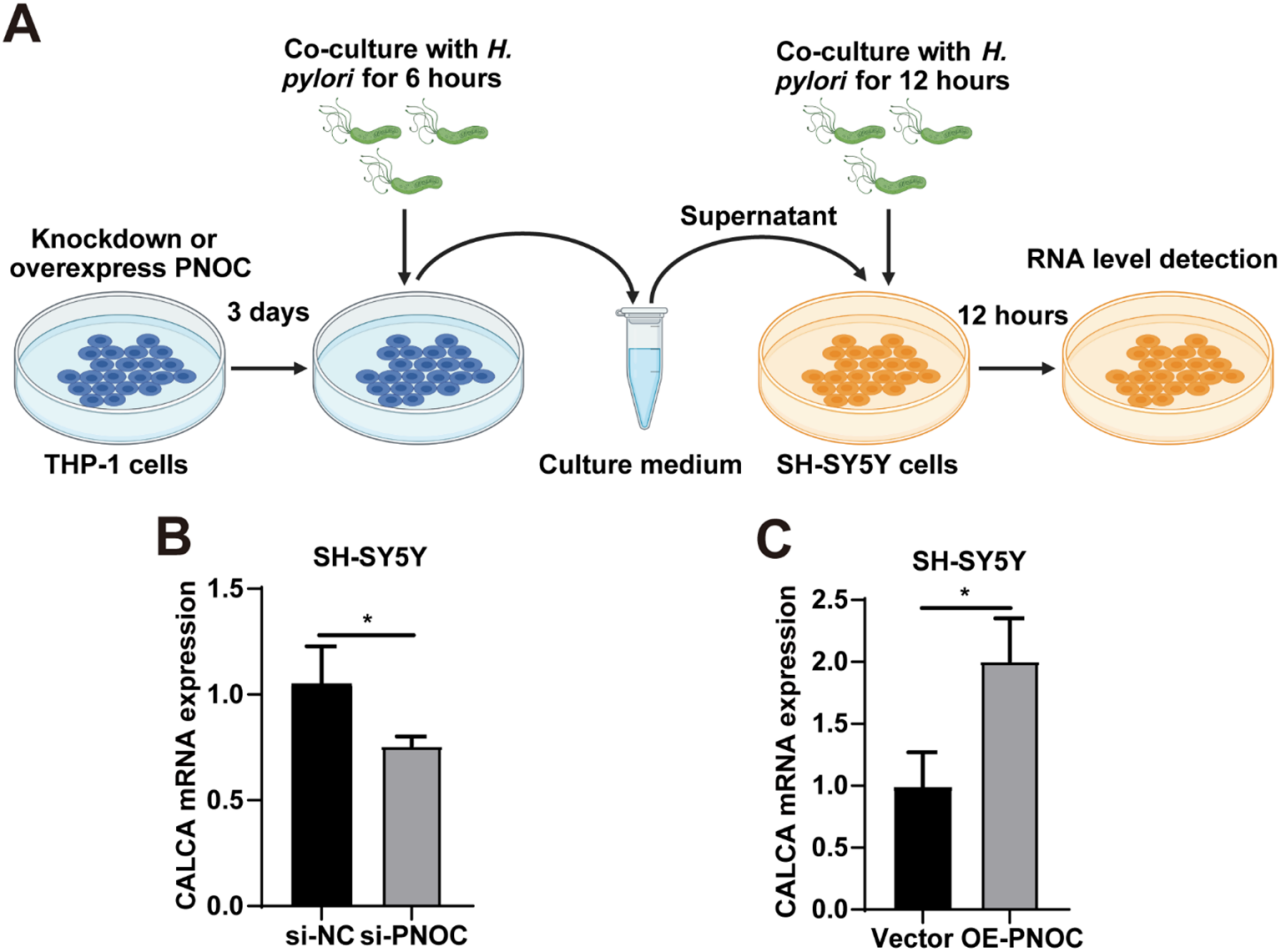
Effect of conditioned medium from PNOC-modulated THP-1 cells on CGRP expression in SH-SY5Y cells. **(A)** Schematic illustration of the experimental workflow: conditioned medium was collected from THP-1 cells following PNOC knockdown or overexpression, and then applied to SH-SY5Y cells. **(B)** Relative mRNA expression levels of PNOC and CALCA in SH-SY5Y cells after incubation with conditioned medium from PNOC-knockdown THP-1 cells, as determined by RT-qPCR. **(C)** Relative mRNA expression levels of PNOC and CALCA in SH-SY5Y cells after incubation with conditioned medium from PNOC-overexpressing THP-1 cells. Data are shown as mean ± SD of n = 3 biological replicates, *P < 0.05, two-tailed unpaired Student’s t test **(B, C)**.

To investigate whether M1-polarized macrophages may influence neuronal gene expression, we employed a co-culture approach to examine the effect of PNOC modulation in THP-1 cells on CALCA expression in SH-SY5Y cells. Conditioned medium from PNOC-knockdown THP-1 cells was associated with reduced CALCA expression in SH-SY5Y cells, whereas medium from PNOC-overexpressing THP-1 cells appeared to promote CALCA expression (**Figures 12A–C**). These results offer preliminary evidence for a neuroimmune mechanism by which *H. pylori* infection may contribute to migraine development, while also providing insights into the molecular basis of its comorbidity with functional dyspepsia.

## 4 Discussion

**Figure 13:**
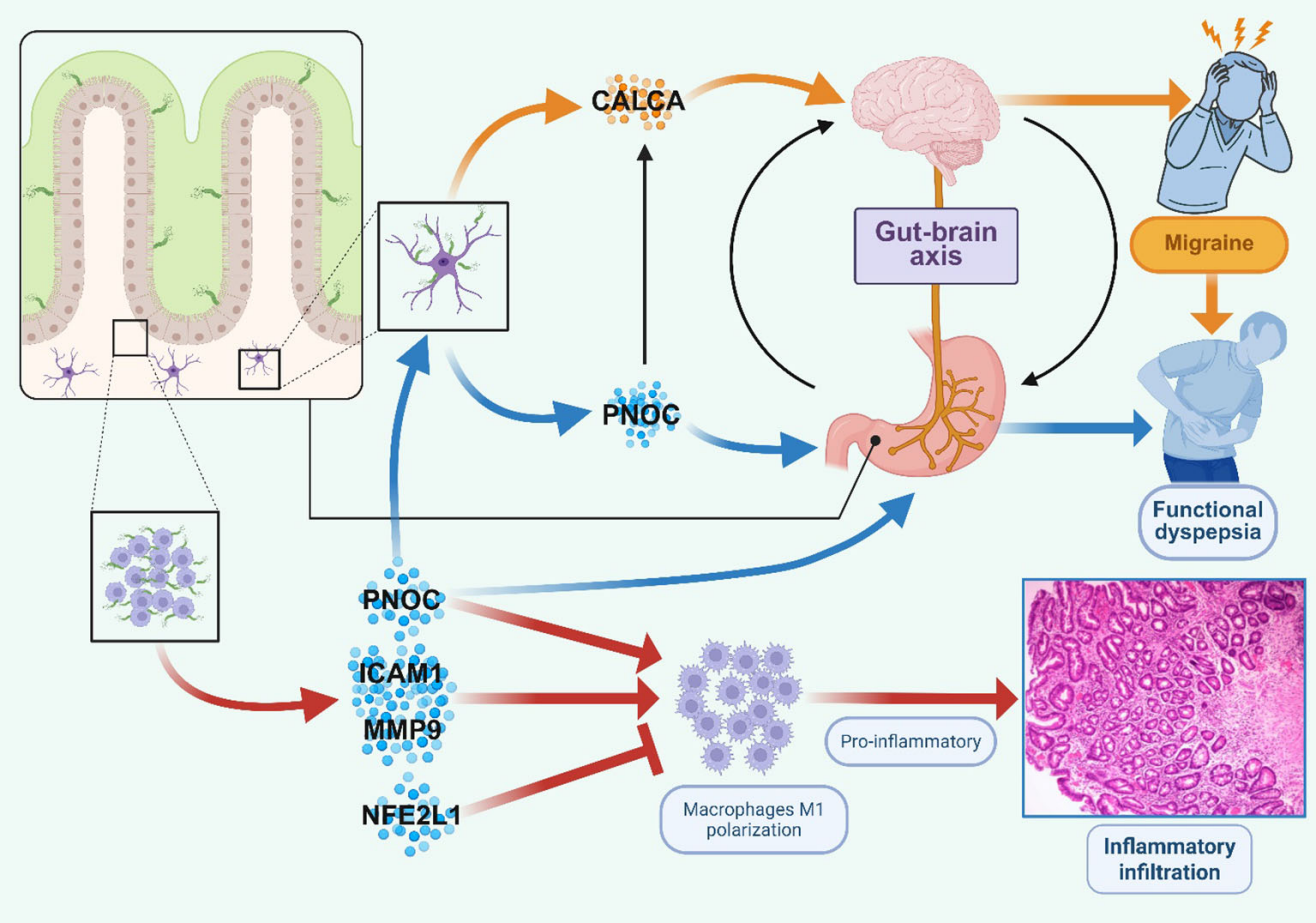
Proposed molecular mechanism by which *H. pylori* induced migraine, functional dyspepsia and inflammation response.

Migraine represents a highly prevalent and disabling neurovascular disorder, affecting approximately 28% of the global population and ranking as the leading cause of neurological disability among individuals aged 5–59 years (4). Clinical observations frequently document the comorbidity of migraine with functional dyspepsia symptoms, including nausea, vomiting, and gastrointestinal dysmotility (5). Despite this established association, the underlying molecular mechanisms connecting these conditions remain poorly characterized. Our study raises the possibility that *H. pylori* infection may serve as a contributing factor in both migraine and functional dyspepsia. Our observations suggest that *H. pylori* infection could exert dual pathogenic effects: (1) through PNOC-CGRP overexpression, potentially activating the gut-brain axis and thereby influencing migraine pathogenesis; and (2) via induction of macrophage M1 polarization and upregulation of PNOC, which may contribute to impaired gastrointestinal motility and functional dyspepsia (**Figure 13**).

Clinical observations have consistently demonstrated a higher prevalence of *H. pylori* infection among migraine patients, with eradication therapy showing significant reductions in migraine-related disability (21, 22). This compelling association prompted our investigation into the potential mechanistic links between *H. pylori* infection and migraine pathogenesis through an integrated bioinformatics and experimental approach. Our comparative transcriptomic analysis identified 683 *H. pylori*-related DEGs that were significantly enriched in several key pathways: Neutrophil chemotaxis and activation, Innate and adaptive immune responses, Inflammatory signaling cascades and Th17, Th1, and Th2 cell differentiation pathways. These findings align with established knowledge of *H. pylori* pathogenesis, wherein the bacterium induces chronic gastritis through neutrophil recruitment and sustained inflammatory responses (55–58). Additionally, the involvement of Th1/Th2 balance regulation suggests a complex immunomodulatory mechanism that could simultaneously facilitate bacterial colonization and promote neurological sensitization (59, 60). This persistent immune activation observed in our analyses may represent a critical link connecting peripheral infection to central nervous system dysregulation, potentially through gut-brain axis communication. Consistent with this notion, previous studies have demonstrated that *H. pylori* infection can contribute to various extra-gastric diseases, including neurological disorders (61, 62). The retrograde axonal transport route along the gut-brain axis has been proposed to play an important role in this process (63, 64). A detailed examination of immune cell populations showed significant changes following *H. pylori* infection, including expansion of γδT cells associated with autoimmune responses, increased B lymphocyte infiltration likely contributing to mucosal defense, and activation of myeloid cells including M0/M1 macrophages and dendritic cell (65–67). Critically, M1 macrophages may establish a gut-brain axis connection by sensitizing meningeal nociceptors through Pannexin 1-dependent mechanisms (36, 37), providing a plausible pathway linking gastric inflammation to migraine pathophysiology. The potential clinical relevance of this mechanism is supported by reports of increase in M1 macrophages in patients with functional dyspepsia (38), raising the possibility that macrophage-mediated neuroimmune crosstalk could represent a unifying mechanism underlying *H. pylori*-associated comorbidities. This dual role may offer a plausible explanation for the observed improvement in migraine symptoms following *H. pylori* eradication, as reduced gastric inflammation might in turn attenuate neuroinflammatory signaling (68).

CGRP is a well-established neurotransmitter in migraine pathogenesis that functions via the gut-brain axis (19). Epidemiological studies have consistently reported a significantly higher prevalence of *H. pylori* infection among migraine patients (18, 69), suggesting a potential link between this bacterial infection and migraine development. Building on these observations, our experimental findings demonstrate that *H. pylori* infection significantly upregulates CALCA (CGRP precursor) expression in neuronal cells, providing direct evidence for *H. pylori*’s capacity to influence migraine through neuronal CGRP signaling along the gut-brain axis. To further explore the molecular mechanisms underlying this association, we employed an integrated approach combining bioinformatics analysis, machine learning, and experimental validation, which led to the identification of four key CGRP-related hub genes—PNOC, ICAM1, MMP9, and NFE2L1—that may mediate *H. pylori*’s effects in this context. PNOC serves as a precursor protein that generates nociceptin and nocistatin, which modulate inflammation through the N/OFQ-NOP pathway (70–72).As a zinc-dependent endopeptidase, MMP9 facilitates inflammatory cell migration and cytokine activation by remodeling extracellular matrix (73). ICAM1 is endothelial adhesion molecule mediating leukocyte extravasation and Th1 immune responses during *H. pylori* gastritis (74–76). Clinical studies confirm that ICAM1 expression decreases following *H. pylori* eradication (77). In contrast, NFE2L1 may function as an anti-inflammatory regulator that modulates inflammatory intensity (50, 78, 79). Consistent with their established roles in immune modulation, PNOC, ICAM1, and MMP9 were positively correlated with M1 polarization markers. Knockdown experiments confirmed that each of these genes contributes to sustaining the pro-inflammatory macrophage phenotype, as their silencing attenuated M1 marker expression. This pro-inflammatory milieu, characterized by sustained M1 polarization, may facilitate bacterial persistence by creating an environment conducive to chronic *H. pylori* colonization. NFE2L1, however, exhibited an opposing regulatory pattern. While bioinformatics analysis showed no significant correlation with immune infiltration, functional experiments revealed that NFE2L1 knockdown enhanced M1 marker expression—consistent with its role as an anti-inflammatory regulator that may limit excessive inflammation (50, 73). This regulatory dynamic—wherein pro-inflammatory mediators (PNOC, ICAM1, MMP9) drive M1 polarization while NFE2L1 tempers the response—may represent a finely tuned host-pathogen equilibrium that allows *H. pylori* to establish chronic infection while avoiding excessive tissue damage. The balance between these opposing forces likely influences not only the persistence of gastric infection but also the extent of local inflammation and, potentially, the likelihood of extra-gastric manifestations.

In addition to its pro-inflammatory effects in macrophages, PNOC was found to regulate neuronal CALCA expression, this suggests that macrophage-derived PNOC may act upstream of CGRP in neuronal cells, modulating expression of this key migraine-associated neuropeptide. Furthermore, nocistatin—a product of PNOC—can directly activate ASIC channels on enteric neurons, impairing gastrointestinal motility (76, 77). Thus, PNOC may serve as a mechanistic link between gastric inflammation and both functional dyspepsia and migraine. Similarly, MMP9 upregulation in *H. pylori* infection has been implicated in migraine and neuroinflammatory processes (80, 81), while ICAM1 inhibition has been associated with migraine prevention (82–84). Together with our findings linking ICAM1 and MMP9 to M1 macrophage polarization, the interconnected roles of PNOC, ICAM1, and MMP9 point to a possible mechanistic framework through which *H. pylori*-associated gastric inflammation could contribute to the development of functional dyspepsia and migraine, offering support for the involvement of gut-brain axis signaling in this comorbidity. Beyond the four hub genes identified in this study, several other CGRP-related genes were also found to be upregulated following *H. pylori* infection, including S100B, CCL2, and TIMP1. These genes have been previously implicated in migraine sensitization and induction (3, 85, 86), suggesting that they may also contribute to the neuroinflammatory processes underlying *H. pylori*-associated migraine. The involvement of multiple CGRP-related genes points to a complex regulatory network through which *H. pylori* infection may influence migraine pathogenesis. Further studies will be helpful to clarify the precise mechanisms by which these genes interact and to better understand their collective role in *H. pylori*-induced migraine.

Nevertheless, this study has certain limitations. The relatively modest sample size and reliance on *in vitro* cell models may not fully capture the complexity of *in vivo* physiological conditions. While our analyses suggest interactions between hub genes and neuroimmune pathways, the precise molecular mechanisms remain to be fully elucidated. Additionally, the cross-sectional nature of transcriptomic data also limits causal inferences. Future studies using animal models, primary human cells, and longitudinal clinical cohorts will be important to validate and extend these findings.

## 5 Conclusion

Taken together, our findings raise the possibility that *H. pylori* infection may contribute to migraine and functional dyspepsia through interconnected mechanisms involving gastric inflammation and neuroimmune signaling. This process is mediated by four key genes: PNOC, ICAM1, and MMP9 appear to promote M1 macrophage polarization and pro-inflammatory responses that sustain gastric inflammation, while NFE2L1 may function as a counterbalancing anti-inflammatory regulator. Among these, PNOC emerges as a potential link between gastric inflammation and extra-gastric manifestations through its effects on gut motility and neuronal CGRP expression. This framework suggests a possible role for gut-brain axis signaling in the comorbidity of migraine and functional dyspepsia, offering insights that could inform therapeutic strategies targeting *H. pylori* eradication and CGRP-related pathways.

## Data Availability

The datasets presented in this study can be found in online repositories. The names of the repositories and accession numbers can be found in the article: The *H. pylori* infection datasets used for the present study(GSE5081, GSE264263, GSE185270, GSE233973, GSE60427) were downloaded from NCBI Gene Expression Omnibus (GEO) database.
