# Supplementary material for: Screening and functional validation of key genes in Helicobacter Pylori-induced macrophage M1 polarization: role in migraine-associated functional dyspepsia
Source: Front Immunol. 2026 Apr 28;17:1684555. doi: 10.3389/fimmu.2026.1684555 (PMC13160760; doi:10.3389/fimmu.2026.1684555)
Supplement: Supplementary file 1 [file Table1.docx]

Supplementary Material


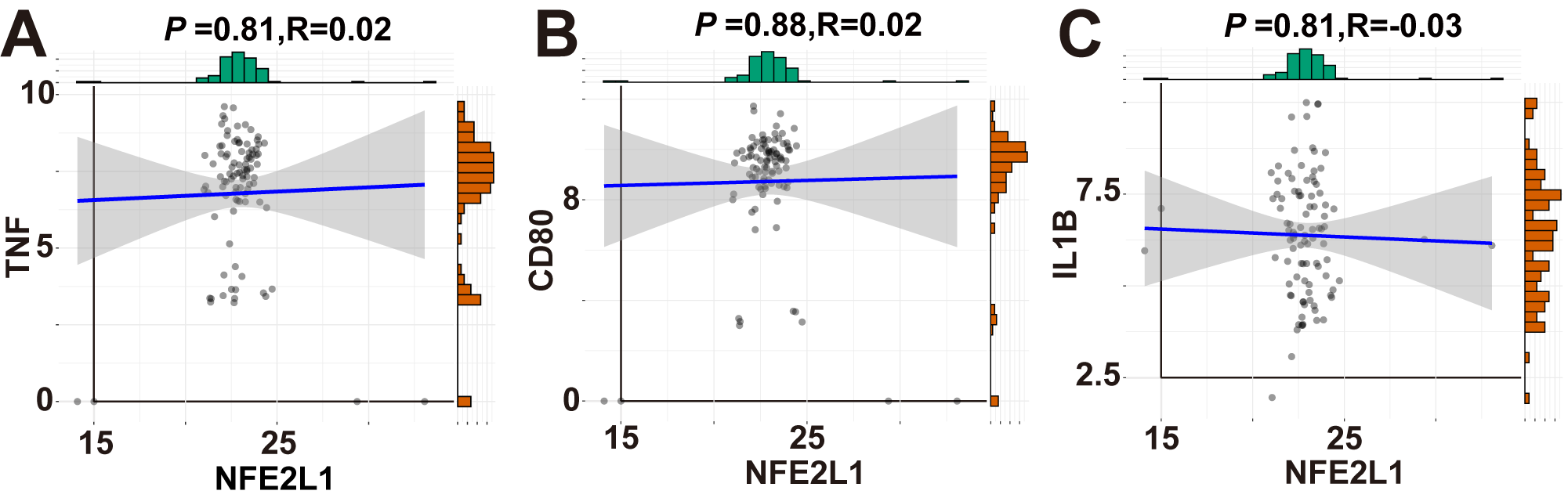


**Supplementary Figure 1** **Correlation analysis between NFE2L1 and inflammatory factors.**

(A) Correlation analysis between NFE2L1 and TNF. (B) Correlation analysis between NFE2L1 and CD80. (C) Correlation analysis between NFE2L1 and IL-1B.


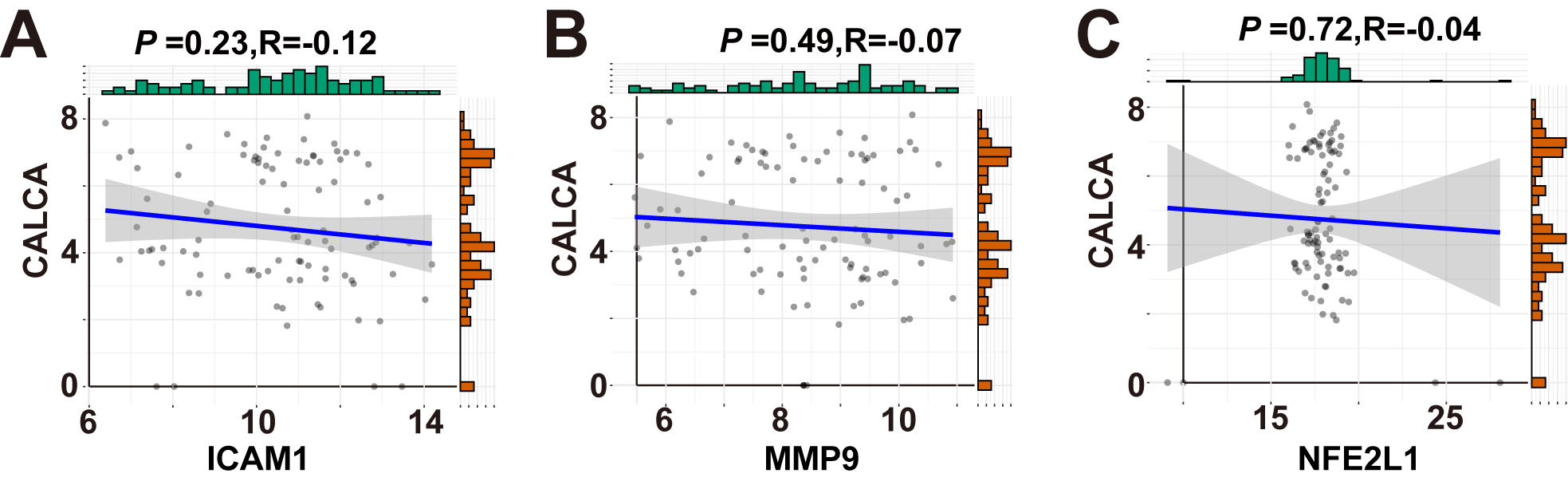


**Supplementary Figure 2 Correlation Analysis of ICAM1, MMP9, NFE2L1 and CALCA.**

(A) Correlation analysis between ICAM1 and CALCA. (B) Correlation analysis between MMP9 and CALCA. (C) Correlation analysis between NFE2L1 and CALCA.
